# Factors related to exclusive breastfeeding in the context of Primary Health Care

**DOI:** 10.1590/2317-1782/20232021163en

**Published:** 2023-10-06

**Authors:** Evelise Rigoni de Faria, Daniel Demétrio Faustino da Silva, Luísa Zadra Passberg

**Affiliations:** 1 Programa de Pós-graduação em Avaliação de Tecnologias para o SUS, Gerência de Ensino e Pesquisa, Grupo Hospitalar Conceição – GHC - Porto Alegre (RS), Brasil.; 2 Residência Multiprofissional em Saúde, Programa Atenção Materno-Infantil e Obstetrícia, Gerência de Ensino e Pesquisa, Grupo Hospitalar Conceição – GHC - Porto Alegre (RS), Brasil.

**Keywords:** Breast Feeding, Primary Health Care, Mental Health, Social Support, Social Conditions, Child Care

## Abstract

**Purpose:**

To identify factors related to exclusive breastfeeding up to the sixth month of life of babies in Primary Health Care services.

**Methods:**

Quantitative, cross-sectional study, carried out with 261 mothers with 12-month-old babies, coming from health units in Porto Alegre. Data related to exclusive breastfeeding, sociodemographic characteristics, maternal mental health, family relationships and childcare follow-up were analyzed using t-tests, chi-square and Poisson regression model.

**Results:**

The rate of exclusive breastfeeding until the sixth month of life was 41%. The mother having a partner increased the rates of exclusive breastfeeding by 46%, while returning to work before six months reduced the chances of exclusive breastfeeding by 31%. No significant association was identified between exclusive breastfeeding and sociodemographic variables, maternal mental health, family relationships, and child health monitoring.

**Conclusion:**

The presence of a maternal partner and the woman's return to work after the baby's six months of life were identified as protectors to exclusive breastfeeding. Health interventions that promote the support network, policies to encourage the practice and transmission of knowledge about breastfeeding are important for better rates of exclusive breastfeeding.

## INTRODUCTION

Breastfeeding favors the initial bond between mother and baby, with repercussions on the child’s development and health in the short and long term. It is considered the isolated strategy that most prevents child deaths worldwide and promotes health to the nursing mother^(^^1^^,^^2^^)^. The World Health Organization (WHO) recognizes exclusive breastfeeding when the child receives only breast milk, without other liquids or solids, except vitamins, oral rehydration salts, mineral supplements, or medications^(^^2^^)^. It is the ideal form of feeding in the baby’s first six months. After this period, it is recommended to maintain complementary breastfeeding until the child is two years old or more ^(^^1^^,^^2^^)^.

According to the Ministry of Health (MS), breast milk has immunological, antimicrobial, and anti-inflammatory properties that reduce the chances of diarrhea and allergies in the child, and hypertension, obesity, and diabetes in the future^(^^1^^)^. The benefits of breastfeeding extend to the nursing mother due to the protective effect against breast cancer, postpartum depression, anxiety, hypertension, diabetes, endometriosis, and osteoporosis. Further to that, there is the fact that breastfeeding does not entail financial costs to the family^(^^1^^,^^3^^)^.

Despite this recommendation, worldwide breastfeeding rates are considered low^(^^4^^,^^5^^)^. Analysis conducted in 80 countries identified a global prevalence of 42% of children up to six months being exclusively breastfed in 2018^(^^4^^)^. In Brazil, in 1986, the prevalence of exclusive breastfeeding until the child is six months old was 2.9%, increasing to 23.9% in 1996, 37.1% in 2006, and decreasing to 36.6% in 2013^(^^5^^)^. Despite the improvement in indicators over the years, they still remain below the recommendations^(^^5^^)^.

Breastfeeding is influenced by several factors, including biological, historical-cultural, economic-social, and psychic, a complex phenomenon and not biologically determined^(^^6^^,^^7^^)^. Sociodemographic characteristics of the mother can contribute to discontinuing exclusive breastfeeding, such as lower age ^(^^8^^,^^9^^)^, low level of education^(^^8^^-^^11^^)^, low family income ^(^^9^^,^^10^^)^, early return to the labor market^(^^8^^-^^10^^,^^12^^)^, absence of a partner^(^^8^^,^^9^^,^^11^^,^^13^^)^, and fewer children^(^^8^^,^^10^^)^. Regarding maternal psychosocial and mental health factors, the literature shows that depressive and anxious symptoms can negatively affect exclusive breastfeeding^(^^14^^)^. Also, lack of family support and conflicting family relationships may constitute an additional stress factor for women and contribute to the interruption of breastfeeding^(^^15^^)^.

The Ministry of Health recognizes the importance of health services in promoting, protecting, and supporting breastfeeding ^(^^1^^)^. Considering that Primary Health Care (PHC) is often the first and main form of access to health care, puerperal monitoring and the growth and development of children (childcare) conducted in health units should address breastfeeding, as well as encourage this practice^(^^1^^,^^11^^)^. On the other hand, failures in health monitoring through childcare or poor quality of consultations are associated with lower rates of exclusive breastfeeding^(^^9^^,^^11^^)^.

Based on the above, this study aimed to identify factors related to exclusive breastfeeding in PHC services. The hypothesis formulated from the data available in the literature is that sociodemographic variables, maternal mental health, satisfaction in family relationships, and health monitoring of children through childcare influence the rates of exclusive breastfeeding.

## METHOD

This is a quantitative, analytical, cross-sectional study derived from child health monitoring cohort projects called “Impact of preventive child oral health programs on Primary Health Care” and “Parental mental health, family relationships, and child health care in early childhood: longitudinal assessment in the first years of life”. These studies involve mothers and children born between 2013 and 2014 from the territory covered by 12 health units attended by the Unified Health System (SUS) in Porto Alegre, RS, Brazil. All children born in this period and followed in the health units when they were one year old were included in the cohorts (n= 765). However, information regarding parental mental health and family relationships was obtained from a subgroup of 261 children and their mothers, whose data make up the sample of this study.

Data collection occurred when the children were one year old through the application of questionnaires to the mothers. The researchers identified the children born between 2013 and 2014 with the teams of the health units and contacted the mothers, presenting the study and the Free and Informed Consent Form. Data collection was conducted in the health units by previously trained researchers during a visit for routine consultation of the children or at another scheduled time with the mothers and involved the following instruments:

*Sociodemographic and health questionnaire:* It investigates demographic and economic data of the family, such as the mother’s age, education, marital status, occupation, income, and housing conditions, as well as data related to the child’s health monitoring in the first year of life (childcare).

*Form of food consumption markers for children - National Food and Nutrition Surveillance System of the Ministry of Health - SISVAN/MS* ^(^^16^^)^: Investigates children’s eating habits from 6 months to 2 years old, including the presence and duration of exclusive breastfeeding.

*Beck Depression Inventory (BDI)* ^(^^17^^)^: Evaluates the presence and severity of depressive symptoms through 21 items. The total score, based on the sum of the item scores, classifies depression in the grades: minimum (up to 11 points), mild (12-19 points), moderate (20-35 points), severe (36-63).

*Beck Anxiety Inventory (BAI)* ^(^^17^^)^: Evaluates the presence and severity of anxious symptoms through 21 items, whose sum of scores classifies anxiety in the minimum (up to 10 points), mild (11-19 points), moderate (20-30 points), or severe (31-63 points) grades.

*Family APGAR* ^(^^18^^)^: Evaluates family functionality through the individual’s responses to five questions they consider: family resources to solve crises; sharing problems and seeking solutions; support throughout the family life cycle; affection between family members; attention to the physical and emotional needs of family members. The total scale score makes it possible to identify: high family dysfunction (0-4 points); moderate family dysfunction (5-6 points); good family functionality (7-10 points).

The data obtained were analyzed in the *IBM SPSS Statistics 22* Program. Associations were made between exclusive breastfeeding up to the baby is six months old (dependent variable) and sociodemographic data variables (age, income, number of children, education, return to work), maternal mental health (depression, anxiety), family relationships, and childcare suitability through bivariate statistical analyses (t and chi-square tests). The Poisson regression model included variables with a p<0.20 coefficient in the bivariate analyses. A 95% confidence interval and 0.05 significance level were considered. The research was approved by the Ethics and Research Committee of Grupo Hospitalar Conceição (GHC) under Opinion No. 13/063 and 2,173,553, and Certificate of Presentation for Ethical Assessment (CAAE) No. 15015013.0.0000.5530 and 69523417.5.0000.5530.

## RESULTS

Data on participants and associations with breastfeeding are in Table 1. Regarding the characteristics of the studied population, we observed a mean maternal age of 28.82 years (7.7). As for family income, 92.34% of the sample was equal to or greater than R$ 700.00/month. The prevailing education among mothers was complete high school (36.78%), followed by incomplete elementary school (18.39%). Most mothers had only one child (55.55%) and lived with a partner (74.33%). When asked about the time to return to work after the child’s birth, 61.30% reported having returned after the full six months of the child or not having worked after birth. Regarding the suitability of childcare follow-up, it was identified that 70.11% of the children had not performed at least seven consultations in their first year of life, a minimum number recommended by the Ministry of Health.

**Table 1:** Data on participants and associations with exclusive breastfeeding up to the children are six months old. Porto Alegre, RS, Brazil. 2019

**Maternal variables**	**Total (n=261)**	**Exclusive breastfeeding until the sixth month**		**p-value**
		**Yes (n=107)**	**No (n=154)**	
Age (mean, standard deviation)	28.82 (± 7.7)	28.96 (± 8.31)	28.69 (± 7.06)	0.780
Family income				
>R$ 700.00	241 (92.34%)	99 (41.08%)	142 (58.92%)	0.780
<R$ 700.00	20 (7.66%)	08 (40%)	12 (60%)	
Education				
Incomplete elementary school	48 (18.39%)	22 (45.83%)	26 (54.17%)	0.489
Complete elementary school	30 (11.49%)	16 (53.33%)	14 (46.67%)	
Incomplete high school	44 (16.86%)	18 (40.91%)	26 (59.1%)	
Complete high school	96 (36.78%)	35 (36.46%)	61 (63.54%)	
Higher education or above	43 (16.47%)	16 (37.21%)	27 (62.79%)	
Number of children				
One child	145 (55.55%)	53 (36.55%)	92 (63.45%)	0.103
Two children or more	116 (44.44%)	54 (46.55%)	62 (53.45%)	
Marital status				
With a partner	194 (74.33%)	87 (44.84%)	107 (55.15%)	0.038
Without a partner	66 (25.29%)	20 (30.3%)	46 (69.69%)	
Return to work				
Returned <6 months	100 (38.31%)	32 (32%)	68 (68%)	0.018
Returned >6 months	160 (61.3%)	75 (46.88%)	85 (53.13%)	
BDI Score*				
Minimum	152 (58.24%)	65 (42.76%)	87 (57.24%)	0.489
Mild	41 (15.71%)	16 (39.02%)	25 (60.98%)	
Moderate	35 (13.41%)	17 (48.57%)	18 (51.43%)	
Severe	24 (9.2%)	07 (29.17%)	17 (70.83%)	
BDI* (mean, standard deviation)	13.07 (± 11.1)	12.21 (± 10.41)	13.68 (± 11.56)	0.301
BAI score┼				
Minimum	138 (52.87%)	58 (42.03%)	80 (57.97%)	0.730
Mild	51 (19.54%)	19 (37.25%)	32 (62.75%)	
Moderate	40 (15.33%)	19 (47.50%)	21 (52.50%)	
Severe	25 (9.58%)	09 (36%)	16 (64%)	
BAI^┼^ (mean, standard deviation)	12.93 (± 12.56)	12.32 (± 11.59)	13.36 (± 13.22)	0.520
Childcare Suitability				
Yes	77 (29.5%)	35 (45.45%)	42 (54.55%)	0.319
No	183 (70.11%)	71 (38.80%)	112 (61.20%)	
Family Apgar (mean, standard deviation)	6.9 (± 3.23)	6.98 (± 3.15)	6.82 (± 3.29)	0.696

* BDI = Beck Depression Inventory;

^┼^BAI = Beck Anxiety Inventory

Concerning maternal mental health, a minimum grade of maternal depression and anxiety was identified, 58.24% and 52.87%, respectively. The rate of women showing symptoms suggestive of depression (moderate to severe intensity) was 22.61%, while 24.91% had anxiety symptoms, also of moderate to severe intensity. The mean total BDI score remained at 13.07 (11.10), indicating mild depressive symptoms, while the mean total BAI score was 12.93 (12.56), corresponding to mild anxiety symptoms. As for satisfaction in family relationships, assessed through the Family Apgar, good family functionality was found for most participants (58.62%), followed by high dysfunction (24.14%) and moderate dysfunction (14.56%), with a mean of 6.90 (±3.23) points on a scale of 0-10, whose higher values indicated better functionality and satisfaction in family relationships.

Regarding the time of exclusive breastfeeding, 14.94% of mothers said they had never breastfed or breastfed less than one month, 4.99% said they had breastfed up to one month, 5.36% up to two months, 7.66% up to three months, 16.10% up to four months, 9.96% up to five months, and 41% up to six full months. All women said they had assistance with child care, with 71.3% citing the child’s father as the main source of support, followed by the child’s grandmother (28.4%).

Associations between the group of women who exclusively breastfed until six months and the group who did not exclusively breastfeed during this period in relation to sociodemographic variables, childcare, maternal mental health, and family relationships identified a statistically significant association between the presence of a maternal partner and the maintenance of exclusive breastfeeding until six months (p=0.038). Similarly, the variable return to work after the baby is six months old (or never having worked) was also significantly associated with the best rates of exclusive breastfeeding as recommended worldwide (p=0.018).

The variables of maternal marital status, time to return to work after the child’s birth, and the number of children were included in the analyses of the multiple model, as shown in Table 2. The findings show that the presence of a maternal partner has a trend of positive association with exclusive breastfeeding up to the child is six months old and may increase the chances of its occurrence by 46%. However, such data should be analyzed with caution, given the marginal p-value identified. Conversely, maternal return to work before the sixth month of life reduces the chances of exclusive breastfeeding by 31% until the baby is six months old.

**Table 2:** Association between maternal marital status, time to return to work, and the number of children with exclusive breastfeeding up to the children are six months old. Porto Alegre, RS, Brazil. 2019

**Maternal variables**	PR*(┼ **95%CI)**	**p-value**
Marital status		
With a partner	1.46 (0.98-2.16)	0.059
Without a partner	1	-
Return to work		
Returned <6 months	0.69 (0.49-0.95)	0.024
Returned >6 months	1	-
Number of children		
Two children or more	1.24 (0.94-1.66)	0.131
One child	1	

* PR=Prevalence Ratio;

^┼^CI = Confidence interval

## DISCUSSION

The data found partially corroborate the hypotheses of the study, considering that associations were identified between the rates of exclusive breastfeeding and the time longer than six months to return to work outside the home, as well as a trend of association with the presence of a partner, reinforcing the importance of the support network for the effectiveness of breastfeeding.

Cautiously, the partner’s presence can be considered protective of exclusive breastfeeding as the data indicated that this factor could increase the chances of exclusive breastfeeding for the recommended time. Similarly, Pereira and collaborators identified in their study that the fact that the woman had a partner increased the prevalence of exclusive breastfeeding by 72%^(^^11^^)^. The maternal perspective highlights the presence of a partner as the most relevant support for breastfeeding^(^^13^^)^. Susin and Giugliani, when investigating the inclusion of partners in intervention to promote breastfeeding, observed that 99.2% of participants wanted to help women in the breastfeeding process^(^^19^^)^. It is known that such help can occur in several ways, directly to the woman, helping her find comfortable positions, supporting her and talking about doubts, fears, and anxieties, and supporting domestic activities and care for other children^(^^9^^)^.

Although approximately 74% of women reported the presence of a partner, it is essential to note that all said they had the help of someone to care for the child, many also mentioning the baby’s grandmother. A review study showed that the fact that women have support from other people influences breastfeeding positively^(^^8^^)^. This also applies to employer support. Osis and collaborators identified that, although women wanted to breastfeed, without support in the workplace, there would be no conditions to maintain exclusive breastfeeding for the recommended time^(^^12^^)^.

Considering that, in Brazil, approximately 25% of the economically active population comprises women with children under six months old, compliance with labor legislation, especially breastfeeding protection laws, becomes fundamental^(^^20^^)^. Brazilian legislation guarantees women contributing to Social Security the benefit of maternity leave, breaks throughout the workday, daycare, and breastfeeding support rooms in the workplace^(^^21^^)^. Despite this, reconciling work and breastfeeding is still challenging, as demonstrated in this study, which identified that maternal return to work before the child is six months old reduces the chances of exclusive breastfeeding.

The literature associates the early return to work with the introduction of other foods and weaning, considering that, on many occasions, women need to carry out their activities away from home, facing long working hours, with little or no flexibility in schedules, also having to reconcile their work activities with other duties, such as household chores^(^^8^^,^^9^^)^. Moreover, there is the aggravating factor of some institutions not fully supporting the practice of breastfeeding or not providing environmental conditions for their workers to breastfeed or milk^(^^8^^)^. In the case of informal workers, who do not have the benefits provided for in the legislation, the situation becomes even more difficult: considering that many are providers of household income or need to supplement it, they return to work early after the child’s birth^(^^22^^)^.

This study supports the literature, which finds that social, economic, emotional, and educational support are essential for exclusive breastfeeding success ^(^^8^^)^. Therefore, the approach to breastfeeding through health education and disseminating knowledge about constitutional rights should be addressed to the general population, not only to pregnant women^(^^12^^,^^20^^)^. Including the family, especially the partner, during consultations with professionals in health services also becomes essential ^(^^9^^)^. Some authors also suggest the readjustment of laws to protect working women who breastfeed so that better institutional support can be provided to allow the continuity of exclusive breastfeeding^(^^12^^)^.

Concerning other sociodemographic data and maternal mental health and family relationships data, contrary to expectations, no significant association was identified with exclusive breastfeeding rates in this study. Similarly, the suitability of childcare follow-up was also not relevant. This situation can be explained, in part, due to the methodological limitations of this research, in which a relatively homogeneous sample was used, of women with good education, with a support network present, universal and free access to health through SUS, living in the same area of the city of Porto Alegre. It is also a limitation of the study that the data collection was conducted when the children were one year old, thus being able to expose results different from those that would be presented when the children were six months old.

Although no association has been identified between maternal mental health and exclusive breastfeeding rates, the percentage found of women presenting depressive and anxious symptoms, 22.61% and 24.91%, respectively, points to the importance of attention to maternal mental health in the first year of life of the child, and to the need for interventions aimed at reducing such mental disorders, which have negative consequences for the mother-infant binomial and also for the family and society^(^^23^^)^. Also, 24.14% of the women presented scores compatible with high family dysfunction, and only 29.5% of the children had the childcare follow-up consultations up to date also deserves attention. In this sense, the need for better investigation of these factors in the study population is reinforced, considering the importance of an effective family and social support network for breastfeeding women.

It is suggested, in order to obtain results of greater statistical relevance in relation to factors related to exclusive breastfeeding, to carry out new studies with a methodology similar to this one; however, to investigate the data in a period close to six months of life of the children, in a more heterogeneous population.

## CONCLUSION

This study found positive evidence regarding the presence of a partner and the maternal return to work outside the home after the children are six months old, considering that these factors contributed to the increase in exclusive breastfeeding rates in the participating population, thus being considered protective factors for breastfeeding, and which should be considered by professionals in PHC. It is believed that this research can contribute to general knowledge regarding the benefits and factors that interfere with breastfeeding and support the formulation of strategies that imply an increase in exclusive breastfeeding rates.
