# Supplementary material for: Factors related to exclusive breastfeeding in the context of Primary Health Care
Source: Codas. 2023 Oct 6;35(5):e20210163. doi: 10.1590/2317-1782/20232021163en (PMC10688288; doi:10.1590/2317-1782/20232021163en)
Supplement: Translation

# Translation: Fatores relacionados ao aleitamento materno exclusivo no contexto da Atenção Primária à Saúde

**DOI:** 10.1590/2317-1782/20232021163pt

**Authors:** Evelise Rigoni de Faria, Daniel Demétrio Faustino da Silva, Luísa Zadra Passberg

**Affiliations:** 1 Programa de Pós-graduação em Avaliação de Tecnologias para o SUS, Gerência de Ensino e Pesquisa, Grupo Hospitalar Conceição – GHC - Porto Alegre (RS), Brasil.; 2 Residência Multiprofissional em Saúde, Programa Atenção Materno-Infantil e Obstetrícia, Gerência de Ensino e Pesquisa, Grupo Hospitalar Conceição – GHC - Porto Alegre (RS), Brasil.

**Keywords:** Aleitamento Materno, Atenção Primária à Saúde, Saúde Mental, Apoio Social, Condições Sociais, Cuidado da Criança

## Abstract

**Objetivo:**

Identificar os fatores relacionados ao aleitamento materno exclusivo até o sexto mês de vida dos bebês em serviços de Atenção Primária à Saúde.

**Método:**

Estudo quantitativo, transversal, realizado com 261 mães com bebês de 12 meses, procedentes de unidades de saúde de Porto Alegre. Dados relativos ao aleitamento materno exclusivo, características sociodemográficas, saúde mental materna, relações familiares e acompanhamento de puericultura foram analisados através de testes t, qui-quadrado e modelo regressão de Poisson.

**Resultados:**

A taxa de aleitamento materno exclusivo até o sexto mês de vida foi de 41%. A mãe ter companheiro aumentou em 46% os índices de aleitamento exclusivo, enquanto retornar ao trabalho antes dos seis meses reduziu em 31% as chances de amamentação exclusiva. Não foi identificada associação significativa entre aleitamento materno exclusivo e variáveis sociodemográficas, saúde mental materna, relações familiares, e acompanhamento de saúde da criança.

**Conclusão:**

A presença de companheiro e o retorno da mulher ao trabalho após os seis meses de vida do bebê foram identificados como protetores ao aleitamento materno exclusivo. Intervenções em saúde que fomentem a rede de apoio, políticas de incentivo à prática e transmissão de conhecimentos sobre amamentação são importantes para melhores índices de aleitamento materno exclusivo.

## INTRODUÇÃO

O aleitamento materno favorece o vínculo inicial entre mãe e bebê, com repercussões no desenvolvimento e na saúde da criança a curto e longo prazo. É considerado a estratégia isolada que mais previne mortes infantis mundialmente, além de promover saúde à nutriz^(^^1^^,^^2^^)^. A Organização Mundial da Saúde (OMS) reconhece como aleitamento materno exclusivo quando a criança recebe somente leite materno, sem outros líquidos ou sólidos, exceto vitaminas, sais de reidratação oral, suplementos minerais ou medicamentos^(^^2^^)^. Consiste na forma ideal de alimentação nos primeiros seis meses do bebê. Após esse período, recomenda-se a manutenção da amamentação, de forma complementar, até os dois anos de vida ou mais^(^^1^^,^^2^^)^.

De acordo com o Ministério da Saúde (MS), o leite materno possui propriedades imunológicas, antimicrobianas e anti-inflamatórias que reduzem chances de quadros de diarreia e alergias da criança, e de hipertensão arterial, obesidade e diabetes no futuro^(^^1^^)^. Os benefícios da amamentação se estendem à nutriz, devido ao efeito de proteção contra câncer de mama, depressão pós-parto, ansiedade, hipertensão, diabetes, endometriose e osteoporose. Soma-se a isso o fato de a amamentação não acarretar custos financeiros à família^(^^1^^,^^3^^)^.

Apesar dessa recomendação, as taxas mundiais de amamentação são consideradas baixas^(^^4^^,^^5^^)^. Análise realizada em 80 países identificou prevalência global de 42% de crianças até seis meses sendo amamentadas exclusivamente no ano de 2018^(^^4^^)^. No Brasil, no ano de 1986, a prevalência de aleitamento materno exclusivo até o sexto mês de vida era de 2,9%, aumentando para 23,9% em 1996, 37,1% em 2006, e diminuindo para 36,6% em 2013^(^^5^^)^. Apesar da melhora nos indicadores ao longo dos anos, os mesmos ainda permanecem aquém das recomendações^(^^5^^)^.

A amamentação é influenciada por diversos fatores, dentre os quais biológicos, histórico-culturais, econômico-sociais e psíquicos, sendo um fenômeno complexo e não biologicamente determinado^(^^6^^,^^7^^)^. Podem contribuir para a descontinuidade do aleitamento materno exclusivo características sociodemográficas da mãe, tais como menor idade^(^^8^^,^^9^^)^, baixo grau de escolaridade^(^^8^^-^^11^^)^, baixa renda familiar^(^^9^^,^^10^^)^, retorno precoce ao mercado de trabalho^(^^8^^-^^10^^,^^12^^)^, ausência de companheiro^(^^8^^,^^9^^,^^11^^,^^13^^)^, e menor número de filhos^(^^8^^,^^10^^)^. Em relação aos fatores psicossociais e de saúde mental maternos, a literatura evidencia que sintomas depressivos e ansiosos podem afetar negativamente o aleitamento exclusivo^(^^14^^)^. Além disso, falta de apoio familiar e relações familiares conflitivas podem constituir um fator de estresse adicional para as mulheres e contribuir para a interrupção da amamentação^(^^15^^)^.

O MS reconhece a importância dos serviços de saúde na promoção, proteção e apoio ao aleitamento materno^(^^1^^)^. Considerando-se que a Atenção Primária à Saúde (APS) é frequentemente a primeira e principal forma de acesso ao cuidado à saúde, o acompanhamento puerperal e do crescimento e desenvolvimento das crianças (puericultura) realizado nas unidades de saúde deve abordar o aleitamento materno, assim como incentivar essa prática^(^^1^^,^^11^^)^. Por outro lado, falhas no acompanhamento de saúde através da puericultura ou má qualidade das consultas estão associadas a menores taxas de aleitamento materno exclusivo^(^^9^^,^^11^^)^.

A partir do exposto, o presente estudo teve como objetivo identificar os fatores relacionados ao aleitamento materno exclusivo em serviços de APS. A hipótese formulada a partir dos dados disponíveis na literatura é de que variáveis sociodemográficas e de saúde mental materna, assim como a satisfação nas relações familiares e o acompanhamento de saúde das crianças através da puericultura influenciem nos índices de aleitamento materno exclusivo.

## MÉTODO

Trata-se de um estudo de caráter quantitativo, analítico, transversal, derivado dos projetos de coorte de acompanhamento de saúde infantil denominados “Impacto de programas preventivos de saúde bucal infantil na Atenção Primária a Saúde” e “Saúde mental parental, relações familiares e atenção à saúde da criança na primeira infância: Avaliação longitudinal nos primeiros anos de vida”. Estes estudos envolvem mães e crianças, nascidas entre 2013 e 2014, procedentes do território de abrangência de 12 unidades de saúde, atendidas pelo Sistema Único de Saúde (SUS) no município de Porto Alegre, RS, Brasil. Todas as crianças nascidas neste período e que seguiam acompanhamento nas unidades de saúde quando estavam com um ano de vida foram incluídas nas coortes (n= 765). Informações relativas à saúde mental parental e relações familiares, no entanto, foram obtidas de um subgrupo de 261 crianças e suas mães, cujos dados compõem a amostra do presente estudo.

A coleta de dados ocorreu quando as crianças estavam com um ano de vida, através da aplicação de questionários às mães. Os pesquisadores identificaram as crianças nascidas entre 2013 e 2014 junto às equipes das unidades de saúde e contataram as mães, apresentando o estudo e o Termo de Consentimento Livre e Esclarecido. A coleta dos dados foi realizada nas unidades de saúde, por pesquisadores previamente treinados, durante visita para consulta de rotina das crianças ou em outro horário agendado com as mães, e envolveu os seguintes instrumentos:

*Questionário sociodemográfico e de saúde:* Investiga dados demográficos e econômicos da família, tais como idade da mãe, escolaridade, situação conjugal, ocupação, renda e condições de moradia, além de dados relativos ao acompanhamento de saúde da criança no primeiro ano de vida (puericultura).

*Formulário de marcadores de consumo alimentar para crianças - Sistema Nacional de Vigilância Alimentar e Nutricional do Ministério da Saúde - SISVAN/MS* ^(^^16^^)^: Investiga hábitos alimentares de crianças de 6 meses a 2 anos, incluindo presença e duração do aleitamento materno exclusivo.

*Inventário Beck de Depressão (BDI)* ^(^^17^^)^: Avalia presença e gravidade de sintomas depressivos através de 21 itens. O escore total, baseado na soma dos escores dos itens, classifica depressão nos graus mínimo (até 11 pontos), leve (12-19 pontos), moderado (20-35 pontos), grave (36-63).

*Inventário Beck de Ansiedade (BAI)* ^(^^17^^)^: Avalia presença e gravidade de sintomas ansiosos através de 21 itens, cuja soma dos escores classifica ansiedade nos graus mínimo (até 10 pontos), leve (11-19 pontos), moderado (20-30 pontos) ou grave (31-63 pontos).

*APGAR familiar* ^(^^18^^)^: Avalia a funcionalidade familiar através das respostas do indivíduo a cinco questões que consideram: recursos familiares para solucionar situações de crise; compartilhamento de problemas e busca de soluções; apoio ao longo do ciclo de vida da família; afetividade entre membros da família; atenção às necessidades físicas e emocionais dos membros da família. A pontuação total da escala possibilita identificar: elevada disfunção familiar (0-4 pontos); moderada disfunção familiar (5-6 pontos); boa funcionalidade familiar (7-10 pontos).

Os dados obtidos foram analisados no Programa *IBM SPSS Statistics 22*. Foram realizadas associações entre aleitamento materno exclusivo até seis meses do bebê (variável dependente) e variáveis de dados sociodemográficos (idade, renda, número de filhos, escolaridade, retorno ao trabalho), saúde mental materna (depressão, ansiedade), relações familiares, e adequabilidade da puericultura através de análises estatísticas bivariadas (testes t e qui-quadrado). As variáveis que apresentaram coeficiente p<0,20 nas análises bivariadas foram incluídas no modelo regressão de Poisson. Foras considerados nas análises intervalo de confiança de 95%, e nível de significância de 0,05. As pesquisas foram aprovadas pelo Comitê de Ética e Pesquisa do Grupo Hospitalar Conceição (GHC) sob Parecer nº 13/063 e 2.173.553, e Certificado de Apresentação para Apreciação Ética (CAAE) nº 15015013.0.0000.5530 e 69523417.5.0000.5530.

## RESULTADOS

Dados das participantes e associações com aleitamento materno encontram-se na Tabela 1. Quanto às características da população estudada, foi observada média de idade materna correspondente a 28,82 anos (7,7). Em relação à renda familiar, para 92,34% da amostra era igual ou superior a R$ 700,00/mês. A escolaridade que prevaleceu entre as mães foi ensino médio completo (36,78%), seguido pelo ensino fundamental incompleto (18,39%). A maior parte das mães possuía apenas um filho (55,55%) e vivia com um companheiro (74,33%). Quando questionadas sobre o tempo de retorno ao trabalho após o nascimento da criança, 61,30% informaram ter retornado após os seis meses completos da criança ou não terem trabalhado após o nascimento. Quanto à adequabilidade do acompanhamento de puericultura, identificou-se que 70,11% das crianças não haviam realizado pelo menos sete consultas no primeiro ano de vida, número mínimo preconizado pelo MS.

**Tabela 1:** Dados das participantes e associações com o aleitamento materno exclusivo até os seis meses de vida das crianças. Porto Alegre, RS, Brasil. 2019

**Variáveis maternas**	**Total (n=261)**	**Aleitamento exclusivo até o sexto mês**		**p-value**
		**Sim (n=107)**	**Não (n=154)**	
Idade (média, desvio-padrão)	28,82 (±7,7)	28,96 (±8,31)	28,69 (±7,06)	0,780
Renda familiar				
>R$ 700,00	241 (92,34%)	99 (41,08%)	142 (58,92%)	0,780
<R$ 700,00	20 (7,66%)	08 (40%)	12 (60%)	
Escolaridade				
Fundamental incompleto	48 (18,39%)	22 (45,83%)	26 (54,17%)	0,489
Fundamental completo	30 (11,49%)	16 (53,33%)	14 (46,67%)	
Médio incompleto	44 (16,86%)	18 (40,91%)	26 (59,1%)	
Médio completo	96 (36,78%)	35 (36,46%)	61 (63,54%)	
Superior ou mais	43 (16,47%)	16 (37,21%)	27 (62,79%)	
Número de filhos				
Um filho	145 (55,55%)	53 (36,55%)	92 (63,45%)	0,103
Dois filhos ou mais	116 (44,44%)	54 (46,55%)	62 (53,45%)	
Situação conjugal				
Com companheiro	194 (74,33%)	87 (44,84%)	107 (55,15%)	0,038
Sem companheiro	66 (25,29%)	20 (30,3%)	46 (69,69%)	
Retorno ao trabalho				
Retornou <6 meses	100 (38,31%)	32 (32%)	68 (68%)	0,018
Retornou >6 meses	160 (61,3%)	75 (46,88%)	85 (53,13%)	
Escore BDI*				
Mínimo	152 (58,24%)	65 (42,76%)	87 (57,24%)	0,489
Leve	41 (15,71%)	16 (39,02%)	25 (60,98%)	
Moderado	35 (13,41%)	17 (48,57%)	18 (51,43%)	
Grave	24 (9,2%)	07 (29,17%)	17 (70,83%)	
BDI* (média, desvio-padrão)	13,07 (±11,1)	12,21 (±10,41)	13,68 (±11,56)	0,301
Escore BAI┼				
Mínimo	138 (52,87%)	58 (42,03%)	80 (57,97%)	0,730
Leve	51 (19,54%)	19 (37,25%)	32 (62,75%)	
Moderado	40 (15,33%)	19 (47,50%)	21 (52,50%)	
Grave	25 (9,58%)	09 (36%)	16 (64%)	
BAI^┼^ (média, desvio-padrão)	12,93 (±12,56)	12,32 (±11,59)	13,36 (±13,22)	0,520
Adequabilidade Puericultura				
Sim	77 (29,5%)	35 (45,45%)	42 (54,55%)	0,319
Não	183 (70,11%)	71 (38,80%)	112 (61,20%)	
Apgar Familiar (média, desvio-padrão)	6,9 (±3,23)	6,98 (±3,15)	6,82 (±3,29)	0,696

* BDI = Inventário Beck de Depressão;

^┼^BAI = Inventário Beck de Ansiedade

No que se refere à saúde mental materna, identificou-se grau mínimo de depressão e de ansiedade maternos, 58,24% e 52,87% respectivamente. O índice encontrado de mulheres apresentando sintomas sugestivos de depressão (intensidade moderada a grave) ficou em 22,61%, enquanto 24,91% apresentaram sintomas ansiosos, também de intensidade moderada a grave. A média de escore total do BDI manteve-se em 13,07 (11,10), indicando sintomas depressivos leves, enquanto a média do escore total do BAI ficou em 12,93 (12,56), que corresponde a sintomas leves de ansiedade. Quanto à satisfação nas relações familiares, avaliada através do Apgar Familiar, constatou-se boa funcionalidade familiar para a maioria das participantes (58,62%), seguido por elevada disfunção (24,14%), e disfunção moderada (14,56%), com uma média de 6,90 (±3,23) pontos em uma escala de 0-10, cujos valores maiores indicavam melhor funcionalidade e satisfação nas relações familiares.

Em relação ao tempo de aleitamento materno exclusivo, 14,94% das mães disseram nunca ter amamentado ou ter amamentado menos de um mês, 4,99% referiram ter amamentado até um mês, 5,36% até dois meses, 7,66% até três meses, 16,10% até quatro meses, 9,96% até cinco meses e 41% até seis meses completos. Todas as mulheres disseram contar com auxílio para os cuidados com a criança, sendo que 71,3% citaram o pai da criança como principal fonte de apoio, seguido da avó da criança (28,4%).

Associações entre o grupo de mulheres que amamentou de forma exclusiva até os seis meses e o grupo que não amamentou de forma exclusiva neste período em relação às variáveis sociodemográficas, de puericultura, saúde mental materna e relações familiares identificaram associação estatisticamente significativa entre a presença de companheiro materno e a manutenção do aleitamento materno exclusivo até os seis meses (p=0,038). De forma semelhante, a variável retorno ao trabalho após os seis meses de vida do bebê (ou nunca ter trabalhado) também esteve associada de forma significativa às melhores taxas de aleitamento materno exclusivo conforme preconizado mundialmente (p=0,018).

As variáveis situação conjugal materna, tempo de retorno ao trabalho após nascimento da criança, e número de filhos foram incluídas nas análises do modelo múltiplo, conforme mostra a Tabela 2. Os achados mostram que a presença de companheiro materno apresenta uma tendência de associação positiva com amamentação exclusiva até os seis meses de vida da criança, podendo aumentar as chances de sua ocorrência em 46%. No entanto, tais dados devem ser analisados com cautela frente ao valor de p marginal identificado. De maneira oposta, o retorno materno ao trabalho antes do sexto mês de vida reduz em 31% as chances de amamentação exclusiva até os seis meses do bebê.

**Tabela 2:** Associação entre situação conjugal materna, tempo de retorno ao trabalho, e número de filhos com o aleitamento materno exclusivo até os seis meses de vida das crianças. Porto Alegre, RS, Brasil. 2019

**Variáveis maternas**	RP*(IC┼ **95%)**	**p-value**
Situação conjugal		
Com companheiro	1,46 (0,98-2,16)	0,059
Sem companheiro	1	-
Retorno ao trabalho		
Retornou <6 meses	0,69 (0,49-0,95)	0,024
Retornou >6 meses	1	-
Número de filhos		
Dois filhos ou mais	1,24 (0,94-1,66)	0,131
Um filho	1	

* RP=Razão de prevalência;

^┼^IC=Intervalo de confiança

## DISCUSSÃO

Os dados encontrados corroboram parcialmente as hipóteses do estudo, tendo em vista que foram identificadas associações entre as taxas de aleitamento materno exclusivo e o tempo superior a seis meses para retorno ao trabalho fora de casa, assim como uma tendência de associação com a presença de companheiro, reforçando a importância da rede de apoio para a efetivação da amamentação.

De forma cautelosa, pode-se considerar a presença do companheiro como protetora ao aleitamento materno exclusivo na medida em que os dados indicaram que este fator pode resultar em aumento nas chances de amamentação exclusiva pelo tempo preconizado. De forma semelhante, Pereira e colaboradores identificaram em seu estudo que o fato de a mulher ter companheiro aumentou a prevalência do aleitamento exclusivo em 72%^(^^11^^)^. A perspectiva materna destaca a presença de companheiro como suporte de maior relevância para a amamentação^(^^13^^)^. Susin e Giugliani, ao investigar a inclusão dos companheiros em intervenção para promover a amamentação, observaram que 99,2% dos participantes desejavam ajudar as mulheres no processo de aleitar^(^^19^^)^. Sabe-se que tal ajuda pode ocorrer de diversas formas, seja diretamente à mulher, auxiliando-a a encontrar posições confortáveis, apoiando-a e conversando sobre dúvidas, medos e ansiedades, e também, no apoio às atividades domésticas e de cuidados com outros filhos^(^^9^^)^.

Apesar de aproximadamente 74% das mulheres terem referido a presença de um companheiro, é importante notar que todas disseram contar com auxílio de alguém para o cuidado com a criança, muitas citando também a avó do bebê. Estudo de revisão demonstrou que o fato de as mulheres possuírem apoio de outras pessoas exerce influência positiva no aleitamento materno^(^^8^^)^. Tal afirmação aplica-se também ao apoio dos empregadores. Osis e colaboradores identificaram que, embora houvesse desejo das mulheres em amamentar, sem a existência de apoio no local de trabalho não haveria condições de manter o aleitamento materno exclusivo pelo tempo preconizado^(^^12^^)^.

Considerando-se que, no Brasil, aproximadamente 25% da população economicamente ativa é composta por mulheres com filhos menores de seis meses de idade, o cumprimento da legislação trabalhista, especialmente às leis de proteção à amamentação, torna-se fundamental^(^^20^^)^. A legislação brasileira garante às mulheres contribuintes da Previdência Social o benefício da licença maternidade, intervalos ao longo da jornada de trabalho, além da existência de creche e salas de apoio à amamentação no local de trabalho^(^^21^^)^. Apesar disso, conciliar trabalho e amamentação ainda é desafiador, conforme demostrado no presente estudo, que identificou que o retorno materno ao trabalho antes dos seis meses de vida da criança reduz as chances de amamentação exclusiva.

A literatura associa o retorno precoce ao trabalho à introdução de outros alimentos e ao desmame, tendo em vista que, em muitas ocasiões, as mulheres precisam exercer suas atividades longe de casa, enfrentando longas jornadas de trabalho, com pouca ou nenhuma flexibilidade em relação aos horários, também tendo que conciliar suas atividades laborais a outras atribuições, como os afazeres domésticos^(^^8^^,^^9^^)^. Além disso, existe o agravante de algumas instituições não apoiarem plenamente a prática do aleitamento materno, ou não fornecerem condições ambientais para que suas trabalhadoras amamentem ou realizem a ordenha do leite^(^^8^^)^. No caso das trabalhadoras informais, que não possuem os benefícios previstos na legislação, a situação fica ainda mais difícil: considerando que muitas são provedoras da renda do lar, ou necessitam complementá-la, retornam ao trabalho precocemente após o nascimento do(a) filho(a)^(^^22^^)^.

Este estudo apoia a literatura, que constata que o apoio social, econômico, emocional e educacional parecem ser imprescindíveis para o sucesso do aleitamento materno exclusivo^(^^8^^)^. Nesse sentido, a abordagem ao aleitamento através da educação em saúde, assim como a disseminação de conhecimentos acerca de direitos constitucionais, deve ser dirigida à população em geral, e não apenas às gestantes^(^^12^^,^^20^^)^. A inclusão da família, especialmente do companheiro, durante as consultas com profissionais nos serviços de saúde também se torna importante^(^^9^^)^. Alguns autores ainda sugerem readequação das leis que se destinam à proteção das mulheres trabalhadoras que amamentam, para que se possa fornecer melhor suporte institucional que permita a continuidade da amamentação exclusiva^(^^12^^)^.

Em relação aos demais dados sociodemográficos e aos dados de saúde mental materna e de relações familiares, ao contrário do esperado, não foi identificada associação significativa com os índices de aleitamento materno exclusivo neste estudo. Da mesma forma, a adequabilidade do acompanhamento de puericultura também não se mostrou relevante. Tal situação pode ser explicada, em parte, devido às limitações metodológicas dessa pesquisa, na qual foi utilizada amostra relativamente homogênea, de mulheres com boa escolaridade, com rede de apoio presente, acesso universal e gratuito à saúde através do SUS, residentes de uma mesma zona do município de Porto Alegre. Surge também como limitação do estudo o fato de a coleta de dados ter sido realizada quando as crianças estavam com um ano de vida, podendo assim expor resultados diferentes dos que se apresentariam aos seis meses de idade das crianças.

Ainda que não tenha sido identificada associação entre saúde mental materna e índices de aleitamento materno exclusivo, o percentual encontrado de mulheres apresentando sintomas depressivos e ansiosos, 22,61% e 24,91% respectivamente, aponta para a importância da atenção à saúde mental materna no primeiro ano de vida da criança, e para a necessidade de intervenções que visem à redução de tais transtornos mentais, que possuem consequências negativas para o binômio mãe-bebê e também para a família e sociedade^(^^23^^)^. Além disso, o fato de 24,14% das mulheres apresentarem escores compatíveis com elevada disfunção familiar, e apenas 29,5% das crianças estarem com as consultas do acompanhamento de puericultura em dia também merece atenção. Nesse sentido, se reforça a necessidade de melhor investigação acerca desses fatores na população do estudo, considerando-se a importância de uma efetiva rede de apoio familiar e social para as mulheres que amamentam.

Sugere-se, para a obtenção de resultados de maior relevância estatística em relação aos fatores relacionados ao aleitamento materno exclusivo, a realização de novos estudos com metodologia semelhante à deste; porém, que investiguem os dados em período próximo aos seis meses de vida das crianças, em população mais heterogênea.

## CONCLUSÃO

Este estudo encontrou evidências positivas quanto à presença de companheiro e o retorno materno ao trabalho fora de casa após os seis meses de vida dos filhos, tendo em vista que tais fatores contribuíram na elevação das taxas de aleitamento materno exclusivo na população participante, sendo assim considerados fatores protetores à amamentação, e que devem ser considerados pelos profissionais na APS. Acredita-se que essa pesquisa possa contribuir para conhecimento geral relativo aos benefícios e fatores que interferem no aleitamento materno, e também para embasamento na formulação de estratégias que impliquem no aumento dos índices de amamentação exclusiva.
